# Decoding of single-trial auditory mismatch responses for online perceptual monitoring and neurofeedback

**DOI:** 10.3389/fnins.2013.00265

**Published:** 2013-12-30

**Authors:** Alex Brandmeyer, Makiko Sadakata, Loukianos Spyrou, James M. McQueen, Peter Desain

**Affiliations:** ^1^Centre for Cognition, Donders Institute for Brain, Cognition and Behaviour, Radboud University NijmegenNijmegen, Netherlands; ^2^Behavioural Science Institute, Radboud University NijmegenNijmegen, Netherlands; ^3^Max Planck Institute for PsycholinguisticsNijmegen, Netherlands

**Keywords:** auditory perception, neurofeedback, EEG, single-trial analysis, multivariate pattern classification

## Abstract

Multivariate pattern classification methods are increasingly applied to neuroimaging data in the context of both fundamental research and in brain-computer interfacing approaches. Such methods provide a framework for interpreting measurements made at the single-trial level with respect to a set of two or more distinct mental states. Here, we define an approach in which the output of a binary classifier trained on data from an auditory mismatch paradigm can be used for online tracking of perception and as a neurofeedback signal. The auditory mismatch paradigm is known to induce distinct perceptual states related to the presentation of high- and low-probability stimuli, which are reflected in event-related potential (ERP) components such as the mismatch negativity (MMN). The first part of this paper illustrates how pattern classification methods can be applied to data collected in an MMN paradigm, including discussion of the optimization of preprocessing steps, the interpretation of features and how the performance of these methods generalizes across individual participants and measurement sessions. We then go on to show that the output of these decoding methods can be used in online settings as a continuous index of single-trial brain activation underlying perceptual discrimination. We conclude by discussing several potential domains of application, including neurofeedback, cognitive monitoring and passive brain-computer interfaces.

## 1. Introduction

The ability to non-invasively measure real-time changes in the patterns of brain activity underlying important perceptual and cognitive processes has led to breakthroughs in areas that were until recently the domain of science fiction. These approaches use systems that analyze neuroimaging measurements (e.g., EEG, MEG, fMRI, PET, fNIRS, etc.) as soon as the necessary data is available, such that results can be used online or in real time. As our understanding of the relationship between various forms of brain activity and specific types of mental states and cognitive processes has grown, so too have the number of potential applications of this knowledge in clinical, medical and educational settings (Sellers and Donchin, 2006; Varma et al., 2008; Sellers, 2013; Tzovara et al., 2013). Here, we present a method for online tracking of brain activity underlying auditory perceptual discrimination. This method is based on the decoding of single-trial auditory evoked potentials, and is illustrated using two datasets collected with variants of the mismatch negativity (MMN) paradigm (Näätänen et al., 2007; Duncan et al., 2009).

In contrast to the averaging methods often used to investigate brain responses measured in EEG, fMRI and other neuroimaging modalities, real-time tracking methods enable researchers to monitor the ongoing dynamics of brain activity as individuals perform different cognitive or behavioral tasks, to use brain responses as a control signal in a brain-computer interface (BCI) setting, or to provide individuals with neurofeedback based on real-time measurements. For instance, real-time fMRI measurements have allowed researchers to develop methods for communication with locked-in patients and patients in vegetative states, as well as novel forms of lie detection and neurofeedback paradigms that help individuals with chronic pain to alleviate some of their symptoms (de Charms, 2008). Others have developed techniques for monitoring working memory function and cognitive load using EEG-based measures (Smith et al., 2001; Brouwer et al., 2012). Single scan dynamic molecular imaging is based on PET measurements and allows for the detection of dopamine release during task performance (Badgaiyan, 2013).

Common to many of these approaches is the use of multivariate pattern classification methods, or so-called decoding approaches (Haynes and Rees, 2006; van Gerven et al., 2009; Blankertz et al., 2011). These machine learning techniques provide a means for making predictions about the mental state of a user on the basis of single-trial neuroimaging data. Predictions are made using a statistical model of a dataset, referred to as a classifier. The dataset used to create the model contains repeated measurements of brain responses corresponding to two or more distinct mental states that are observed in a given task setting. The classifier is trained to identify specific features in the data (e.g., fMRI voxels in regions of interest, EEG samples at specific channels and time-points) that provide discriminative information about the distinct classes of mental states that have been defined as part of the classification problem. Once trained, a classifier can make predictions about novel, previously unseen data.

A clear example of this type of classification problem is provided by BCI systems that use EEG measurements and paradigms designed to elicit the P300 response [for a review of the event-related potential (ERP) literature, see Polich (2007), for P300-based BCIs, see Farwell and Donchin (1988), Nijboer et al. (2008), Schreuder et al. (2010), Belitski et al. (2011), van der Waal et al. (2012)]. The P300 response is elicited using sequences containing a rare target event randomly embedded in a series of non-target events. The presentation of a target will draw the user's attention, and is reflected in the P300 component. Data collected in this paradigm can be thought of as belonging to two classes: targets and non-targets. As the P300 response is only elicited by targets, data collected on target trials will contain the P300 response while non-target trials will not. Given sufficient amounts of data, a classifier trained on such a dataset will learn to assign importance to specific features of the data corresponding to P300 responses elicited in individual trials while ignoring other features unrelated to the two classes of interest. Such a classifier can be used in various BCIs, such as those implementing communication devices or menu systems.

**Figure 1:**
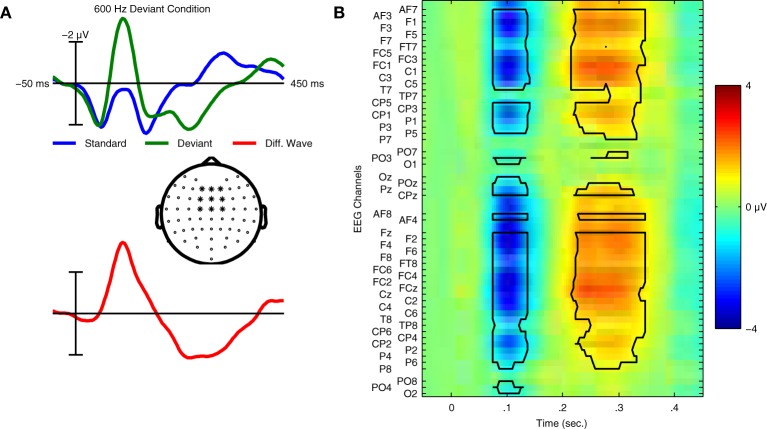
**Grand average ERPs and statistical analysis for Dataset 1. (A)** Grand average ERPs of 14 participants in Dataset 1 for both standard and deviant trials are shown with the deviant minus standard difference wave used to quantify the mismatch negativity response. ERPs were calculated using the average of 9 fronto-central electrode locations (indicated in the scalp map) where the MMN response is typically maximal in amplitude. Relative to the ERP for the standard trials, the deviant ERP shows an enhancement of the negative peak in the N1 time window and an additional positivity between 200 and 300 ms relative to stimulus onset. This component is referred to as the P3a, and is elicited in MMN paradigms that employ large stimulus contrasts. **(B)** Statistical analysis of the group level responses. Grand average ERPs for all 14 participants were analyzed using a non-parametric cluster randomization procedure (Maris and Oostenveld, 2007) across all 64 EEG channels for time points between 80–350 ms relative to stimulus onset. The results of this analysis are presented using an image of the grand-average difference wave. Two significant clusters of activity (outlined in black) were found, corresponding to the MMN (*p* < 0.001) and P3a (*p* < 0.001) responses. The brain responses underlying these components represent two stages in the automatic sensory discrimination process triggered by the presentation of the deviant stimulus.

In the context of auditory perception, similar sequences are used to elicit another ERP component: the MMN response. These “oddball” sequences contain frequent standard trials and rare deviant trials, each corresponding to a different type of sound. For example, the standard sound might be a musical tone with a specific fundamental frequency (*f*0), while the deviant sound has a different *f*0. Whereas the P300 response is elicited using an active task (attend to targets), the MMN is elicited without attending to the stimuli; participants instead watch silent films while oddball sequences of sounds are presented. In the auditory ERP, the MMN is usually maximal at fronto-central electrode locations and peaks between 100 and 300 ms, depending on the type of stimuli employed (Näätänen et al., 2007). Examples of the ERPs elicited in an MMN paradigm can be found in Figure 1.

Interestingly, the MMN response reflects individual differences in perceptual discrimination abilities, such as those related to native-language background (Naatanen et al., 1997; Winkler et al., 1999; Brandmeyer et al., 2012) and the effects of musical training (Koelsch et al., 1999; Fujioka et al., 2004), as well as the longitudinal effects of perceptual learning (Tremblay et al., 1997; Menning et al., 2000). Abnormalities in the MMN response are associated with different clinical and medical conditions, such as developmental disorders (Bishop, 2007), schizophrenia (Michie, 2001) and coma (Fischer et al., 2004). This diagnostic aspect of the MMN has led to an interest in the use of decoding methods to analyze single-trial MMN responses. For instance, it has been shown that decoding performance reflects differences in categorical speech perception by native and non-native speakers (Brandmeyer et al., 2013), and that decoding analyses can be used to predict survival rates in comatose patients (Tzovara et al., 2013).

The ability to track ongoing MMN responses in real-time can provide novel insights into the dynamic nature of perceptual processes. Specifically, real-time monitoring of MMN responses would provide insights into both short- and long-term changes in brain responses associated with perceptual learning. Furthermore, the same real-time tracking technique could serve as the basis of a neurofeedback paradigm centered on auditory perceptual learning, by providing users with ongoing feedback on brain responses associated with discrimination sensitivity. The remainder of this article defines a real-time tracking approach based on single-trial decoding of auditory evoked potentials using a logistic regression classification algorithm. First, important aspects of the decoding approach are illustrated, including an outline of the pattern classification problem, data preprocessing, feature evaluation and the generalization of classifier performance. Then a method for interpreting the online classifier output as a continuous signal for use in real-time applications is presented.

Two datasets are used throughout to illustrate various aspects of the decoding and real-time tracking method: one collected using a standard MMN oddball paradigm (Näätänen et al., 2007; Duncan et al., 2009), and one collected using a so-called optimal MMN paradigm (Naatanen et al., 2004). All data were collected using a passive listening task in which participants viewed silent films while auditory stimuli were presented. For both datasets, simple tone stimuli were used to elicit the MMN response. Such stimuli are widely used in auditory research, as well as in MMN research. Such tones elicit ERPs containing components whose timing and spatial topography are well understood. A summary of the two datasets can be found in Table 1. A complete description of the methods used to collect these datasets is provided in the supplementary materials.

**Table 1:** **Summary of Datasets 1 and 2**.

**COMMON RECORDING METHODS**	
EEG System	Biosemi Active 2 amplifier with 64 EEG channels w/ horizontal and vertical EOG, left and right mastoid leads. Data recorded at a sample rate of 2048 Hz and downsampled offline to 256 Hz.
Stimulus presentation	Etymotic ER-4P insert headphones calibrated to approximately 70 dB SPL
Data epochs	Data epoched between −200 and 600 ms (relative to stimulus onset) for preprocessing, data between −50 to 450 ms (non-overlapping epochs) used for ERP and classification analysis.
**DATASET 1**	
Participants	14 normal hearing adults
Stimuli	Pure sinusoidal tones at 500 Hz (standard) and 600 Hz (deviant), 100 ms duration
Sequence design	Oddball sequences with 85% standard stimuli, 15% deviant stimuli, interstimulus interval of 500 ms, 1000 total trials per block (150 deviants).
	Based on Duncan et al. (2009).
No of blocks per session	2
No of sessions	1
**DATASET 2**	
Participants	12 normal hearing adults
Stimuli	Harmonic sinusoidal tones at 500 Hz, 75 ms duration with the following deviant stimuli: Location (±800 μS inter aural delay), Frequency (10% increase in *f*0), Amplitude (±10 dB), Duration (25 ms) and Gap (insertion of 25 ms of silence).
Sequence design	Optimal MMN sequences with 50% standard stimuli and 10% of each of the five types of deviant, alternating standard and deviant stimuli, interstimulus interval of 500 ms. 600 total trials per block (300 deviants). Based on Naatanen et al. (2004), Duncan et al. (2009).
No of blocks per session	3
No of sessions	3

## 2. Single-trial decoding of auditory ERPs containing the MMN response

**Figure 2:**
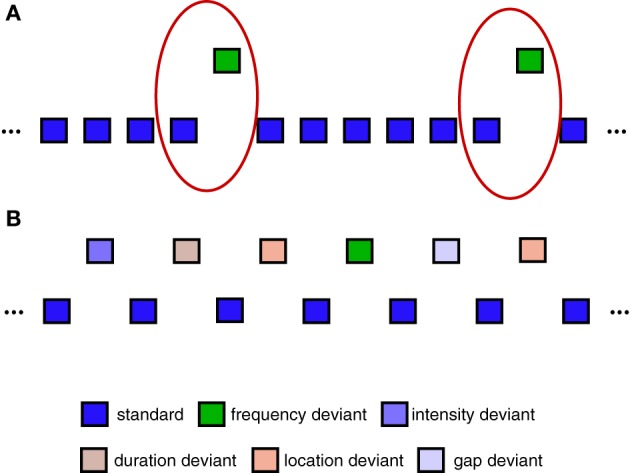
**Sequences used during data collection. (A)** Example of oddball sequences used to collect Dataset 1. An oddball sequence with a 500 Hz standard stimulus (85% of trials) and a 600 Hz deviant stimulus (15% of trials) was used to collect Dataset 1. Only data from deviant trials and the standard trials immediately preceding them were used in the classification analyses (illustrated using red circles). Each block contained a total of 1000 trials. **(B)** Example of “optimal” MMN sequences used to collect Dataset 2. An alternative sequence structure utilizing 5 different types of deviant stimuli can be used to reduce the overall amount of time required to collect MMN responses (Naatanen et al., 2004; Duncan et al., 2009). Here, all standard and deviant trials are utilized in the classification analysis. Each block contained a total of 600 trials.

The use of pattern classification to decode single-trial EEG data containing different types of task-related brain responses is a hallmark of non-invasive BCI systems (van Gerven et al., 2009) and EEG-based decoding analyses (Schaefer et al., 2011; Brandmeyer et al., 2013). Single-trial analysis and classification of ERP components can be understood in terms of spatial and temporal patterns in the data that are associated with one or more components (Blankertz et al., 2011). Here, we focus specifically on a binary classification problem in which the aim is to predict whether a specific single-trial ERP measurement represents a standard (no MMN) or deviant (MMN) trial. The oddball sequence used to collect Dataset 1 is illustrated in Figure 2A. Only standard trials which immediately preceded a deviant trial were selected for inclusion in the classification analysis. This balanced the amount of data for each of the two trial types, and is the same as the approach taken in Brandmeyer et al. (2013). More recent MMN studies have made use of an “optimal” MMN paradigm, which employs a sequence structure containing multiple deviant stimuli, and which is illustrated in Figure 2B. Dataset 2 was collected using this type of sequence. As an equal number of standard and deviant stimuli are presented, all trials were utilized in the analysis.

The data epoch associated with each trial can be represented as an *m* * *n* matrix, where *m* is the number of EEG channels that are measured (the spatial dimension) and *n* is the number of recorded samples (the temporal dimension). The data presented here were collected using *m* = 64 EEG channels and *n* = 128 samples, representing data collected between −50 and 450 ms relative to stimulus onset at a sample rate of 256 Hz. Given a labeled dataset (typically “−” and “+”) containing examples of standard and deviant trials, a classifier is trained such that each dimension of the corresponding data is assigned a weight, stored in an *m* * *n* weighting matrix *w*. The weights are adjusted on the basis of the training data with respect to differences in the observed spatio-temporal patterns associated with the two types of trials, while also taking into account variance in the data not associated with either of the two classes. This latter aspect of the classification problem is crucial in determining the performance of the analysis, given the low signal to noise ratio of MMN measurements [approximately 1–5 μV for the MMN response compared to ongoing EEG activity, which can exceed ±30 μV, Handy (2005), Duncan et al. (2009)].

The method used to obtain the weights *w* determines the type of classifier which is obtained. Methods such as linear discriminant analysis (LDA), support vector machines (SVM) and logistic regression represent just a few of the most common algorithms used. Additionally, the classifier is said to be either linear or non-linear. The choice to use a linear or non-linear classifier is often based on the amount and type of data being analyzed, with linear algorithms often preferred for their simplicity (Muller et al., 2003). In the present analysis, which aimed at continuous monitoring of EEG signals, we chose to make use of a linear logistic regression algorithm for a number of reasons. Firstly, logistic regression classifiers have been shown to provide relatively high performance for EEG data in the context of BCI applications (Farquhar and Hill, 2013). Secondly, the output of logistic regression classifiers can be interpreted probabilistically, as opposed to the output of SVMs and other classifier families (Bishop, 2009). This enables a distinct set of applications which are discussed later.

Given a trained classifier with weights *w* and an epoch of data *x*, both of which are vectorized versions of the corresponding matrices, the output of a linear classifier is calculated as follows:

(1) $$f(x)=w^{⊤}x+b$$

where *b* is a bias term. This output is referred to as a “decision value.” For logistic regression classifiers, the range of *f*(*x*) is (−∞, ∞), with the sign of *f*(*x*) representing a prediction about which of the two classes in the binary problem *x* belongs to. A classifier's performance is determined by quantifying the accuracy of predictions it makes about previously unseen data. In other words, we want to know how well a classifier will generalize to novel situations, such as the real-time applications discussed in the introduction. To determine generalization, a given dataset is typically split into two subsets: a training set and a test set. The training set (also referred to as a calibration set) contains data that are used to construct the classifier, while the test set is used to determine how accurately the classifier can make predictions about novel exemplars; because the labels have been provided for epochs in the test set, classifier predictions can be compared with the true class of the individual exemplars, and the percentage of correct decisions can be calculated. This is referred to as the classification rate.

Rather than estimating generalization using a single training and test set, cross-validation methods can be used. Following the creation of *x* partitions of the dataset, *x* iterations of the classification analysis are carried out. In each iteration, a distinct set of *x* − 1 partitions are used as the training set while the remaining partition is used as a test set. Each of the partitions is thus used as a test set in one of the analyses. The classification rates presented here reflect the results of a 10-fold cross-validation procedure, unless otherwise specified. Thus, in each of the 10 iterations, 90% of the available data is used for training, while the remaining 10% is used for testing. The final classification rate obtained in such an analysis, referred to as a cross-validation rate, is the mean of the classification rates obtained for each of the 10 test sets.

An important relationship is defined between the classification rate and the number of classes in a given classification problem. In the case of a binary classifier, imagine that, instead of a classifier, we merely had a random number generator spitting out 1 s and 0 s (i.e., “+” and “−,” the labels of each of the two classes) and making predictions about our data. This is equivalent to the problem of predicting a fair coin toss. In both cases, given infinite trials, we would expect correct answers 50% of the time. This value is defined as chance performance. A binary classifier performing at chance level is essentially no more useful than a random number generator or flipping a coin.

In real-world classification problems, it is rarely possible to achieve 100% classification accuracy. However, poorly defined classification problems can lead to chance-level performance. It is possible to determine whether the results of a given binary classification analysis are significantly different from chance-level performance using binomial confidence intervals (Müller-Putz et al., 2008; Pereira et al., 2009). This is, for instance, the same manner in which the error of a poll is determined, and is based on the number *k* of observations that are available. A confidence interval *I* is defined around a specific value *p* (in the case of a binary problem, chance-level performance, where *p* = 0.5) as follows:

(2) $$I=p\pm z_{1}-\frac{1}{2}\alpha \sqrt{\frac{1}{k}p(1-p)}$$

where $z_{1}-\frac{1}{2}\alpha$ represents a z-scored percentile from a normal distribution for a specific error-value α. Importantly, this implies that the confidence interval becomes smaller as *k* grows. For example, for a statistical confidence level of α = 0.05, the confidence interval for 10 observations is ±25.1%, but is only ±9.6% for 100 observations and ±3.1% for 1000 observations. The observation of classification rates significantly above chance level implies that predictions made by the classifier are non-random. This in turn suggests that the classifier training data contains information that is useful for distinguishing new examples of the different classes from one another, even if this information is not completely reliable.

An alternative to the binomial confidence interval is the permutation test, which can be used in cases where the assumption of class independence does not hold, or when there is reason to suspect bias. Rather then testing the null hypothesis using the binomial distribution, the observed results are compared to the distribution obtained by repeatedly permuting the true class labels belonging to the data and recalculating the classification performance. The probability of the null hypothesis is then calculated as the proportion of the resulting distribution with classification performance greater than or equal to the observed result Pereira et al. (2009). In general, however, the significance of classification results presented here are based on the use of binomial confidence intervals, assuming chance-level performance of 0.5.

A potential problem arising in classification analyses is the over-fitting of the classifier to the training data. What this means is that the model does not generalize well to new examples outside the training set. This is especially troublesome for high-dimensional data sets such as those encountered in neuroimaging. For instance, the present data contains 64 × 128 = 8192 dimensions. A rule-of-thumb in pattern classification is that the number of examples needed to train a classifier is roughly equal to the number of dimensions in the data (Duda et al., 2001; Blankertz et al., 2010). However, this is very often impractical in the case of neuroimaging data due to the amount of time required to obtain suitable training data.

One solution for dealing with over-fitting is the use of regularization methods. These methods limit the complexity of classifier models, as over-fitting is associated with relatively more complex models. For logistic regression classifiers, the level of regularization is a function of the total variance observed in the training data. A weighting parameter *c* determines classifier complexity, with smaller values leading to more complex models. The optimal value of *c* is determined using a grid search with the values [0.001, 0.01, 0.1, 0, 1, 10, 1000], respective to the overall variance of the data: the 10-fold cross-validation procedure is repeated for each value of *c*, and the model trained with the regularization setting leading to the highest overall performance is selected.

A principal requirement of classifiers intended for use in real-world applications is that their performance should be as high as possible. Prior to classifier training, pre-processing and/or feature selection steps are typically performed. These steps aim to remove noise from the data and to reduce its dimensionality. The precise nature of the steps taking during pre-processing and feature selection is determined by the type of data. Additionally, the amount and type of data available for classifier training can significantly impact their performance.

To summarize, the present approach uses pattern classification methods to make predictions about single-trial auditory ERPs collected in an auditory mismatch paradigm. Specifically, we use 500 ms epochs of 64-channel EEG data from two types of trials, standard and deviant, to define a binary classification problem. We then train a regularized, linear logistic regression classifier using equal amounts of data from both types of trials. A 10-fold cross-validation is used initially to estimate generalization performance. The following sections present a data-driven approach in which preprocessing parameters for classification of MMN responses are optimized, and examine the effects of training set size and data selection on classifier performance. Relative changes in classifier performance between analysis steps are evaluated using paired-samples *t*-tests. Methods for evaluating data features that influence classification performance are also presented, followed by an assessment of generalization performance using datasets collected from different measurement sessions or individuals.

### 2.1. Pre-processing

In the case of EEG data, pre-processing is also a typical step in ERP analyses, and many of the specific actions taken, such as artifact removal, filtering and resampling, remain the same. However, due to the nature of the pattern classification problem, the parameters used during these steps may differ from those employed in ERP paradigms. For instance, the cutoff frequencies and downsampling employed by decoding approaches are often more severe due to the influence of noise and brain activity unrelated to the primary task on classifier performance. Recent work has evaluated pre-processing methods for obtaining optimal classification performance with EEG data collected in different BCI paradigms (Farquhar and Hill, 2013). We perform a similar evaluation, and show that the optimal parameters are somewhat different for data collected using an MMN paradigm.

The datasets presented here were initially preprocessed using the following steps: (1) Bad channels (i.e., those with offsets greater than 35 mV or with 50 Hz power greater than 1000 μV^2^) were repaired using a spherical spline interpolation procedure (Perrin et al., 1989) on a per epoch basis. This has the effect of removing artifactual and noise-related activity that can influence classification performance, and preserves the full electrode montage for subsequent steps in the decoding analysis. (2) An independent component analysis was performed, and components containing artifactual activity were selected using a threshold calculated on the basis of the mean variance across trials and components. These selected components were removed from the data, and the remaining components were reprojected onto the original EEG recording channels. The same method has been employed in Bishop and Hardiman (2010) and Brandmeyer et al. (2013) in the context of MMN paradigms, and was originally described in Jung et al. (2000). This has the effect of removing eye movements and other muscular artifacts that would otherwise lead to the rejection of data epochs in subsequent steps. The preservation of individual epochs is important for optimizing performance, as increased training set size leads to higher classifications rates. (3) Data were band-pass filtered between 1 and 25 Hz (Kujala et al., 2007). (4) Individual epochs were inspected at all EEG channels for activity exceeding ±75 μV, in which case they were excluded from subsequent analyses. (5) Lastly, data were re-referenced to the average of the two mastoid leads, which is known to enhance the signal-to-noise ratio of MMN responses (Kujala et al., 2007; Duncan et al., 2009).

**Figure 3:**
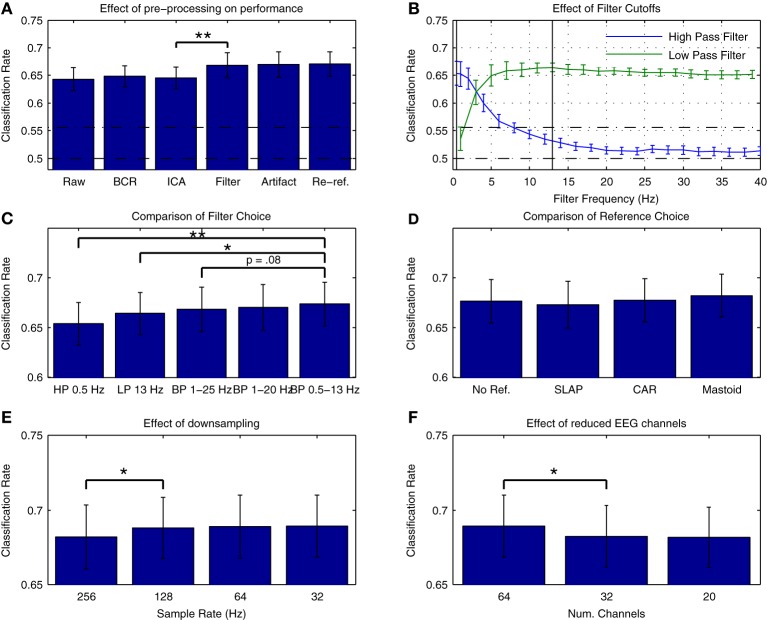
**Effects of preprocessing on cross-validation performance for Dataset 1. (A)** Comparison of average cross-validation rates across subsequent stages of preprocessing. Individual participant's data were used to train a linear logistic regression classifier following each of five preprocessing steps: bad channel repair (BCR), independent component analysis based artifact removal (ICA) (Bishop and Hardiman, 2010), band-pass filtering between 1 and 25 Hz [as suggested in (Kujala et al., 2007)], rejection of data epochs containing activity exceeding ±100 μV, and re-referencing the data to the average of the two mastoid leads (Kujala et al., 2007; Duncan et al., 2009). Baseline correction between −50 and 0 ms (relative to stimulus onset) was applied at each step. As is illustrated, a significant increase in performance was obtained during the filtering stage. **(B)** Evaluation of filter cutoff frequencies. Two separate grid search procedures were used to evaluate the effect of high- and low-pass cutoff frequencies on performance. Optimal cutoffs of 0.5 and 13 Hz were found for the low- and high-pass filters, respectively, and are illustrated in the plot. **(C)** Comparison of filters. Classification rates using the optimal low- and high-pass filter parameters as well as the original 1–25 Hz band-pass filter (Kujala et al., 2007), a 1–20 Hz band-pass filter as recommended by Duncan et al. (2009) and a 0.5–13 Hz band-pass filter based on the previous analysis are shown. Performance was highest using the 0.5–13 Hz band-pass filter. **(D)** Comparison of reference choice. Cross-validation rates are shown for unreferenced data as well as for three possible reference choices: common-average reference (CAR), a surface laplacian reference (Farquhar and Hill, 2013) and a mastoid reference. Although no significant difference in classifier performance was found, performance was highest overall when using a mastoid reference. As has been previously discussed in the literature, the use of a mastoid reference can enhance the signal-to-noise ratio of auditory ERPs collected using an MMN paradigm (Kujala et al., 2007; Duncan et al., 2009). **(E)** Effect of downsampling on cross-validation rates. Classifier performance using the original 256 Hz sampling rate and the updated filter cutoffs was compared to performance using data resampled at 128, 64, and 3 Hz. A significant improvement in average performance was observed for the initial step, with the highest performance observed for data downsampled to 32 Hz. As the high-pass cutoff of 13 Hz is below the nyquist frequency (approximately 16 Hz), these improvements suggest that the removal of unnecessary dimensions in the data has a beneficial effect on classifier performance. **(F)** Effect of reduced EEG channels. A comparison of the initial 64-channel montage with 32- and 20-channel montages based on the international 10–20 system showed a significant decrease in performance. This implies that features of the data related to the topography of the EEG signal have an important bearing on classification performance. For all subfigures, the significance of paired-sample *t*-test comparisons is indicated using asterisks: ^*^p < 0.05, ^**^p < 0.01.

Figure 3A presents average cross-validation rates obtained with Dataset 1 when training classifiers on individual datasets following each preprocessing step. Significant improvements in performance were obtained when the data were filtered [*t*_(13)_ = −3.026, *p* = 0.01]. Are additional improvements possible through more refined selection of cutoff frequencies? Figure 3B presents an assessment of the effects of high- and low-cutoff frequencies. Maximal performance was obtained with high- and low-pass frequencies of 0.5 and 13 Hz, respectively. A comparison of performance using different filters is presented in Figure 3C. Performance was slightly higher relative to the original 1-25 Hz band-pass filter when using a 0.5–13 Hz band-pass filter, as well as relative to a 1–20 Hz band-pass filter as recommended for MMN recordings in Duncan et al. (2009). Research investigating the oscillatory activity underlying the time-domain MMN response has revealed that phase resetting and power modulation of theta band activity (4–8 Hz) in the temporal and frontal MMN generators, respectively, occur during deviant trials (Fuentemilla et al., 2008). This suggests that activity in and around this frequency band will provide discriminative information for the present classification analysis. Other studies classifying MMN data (Herrmann et al., 2012) and auditory evoked responses to music (Schaefer et al., 2011) have also made use of similar filter settings during preprocessing, indicating that band-pass filtering in the 0.5–13 Hz range might improve decoding performance for auditory brain responses.

The choice of reference electrodes represents another preprocessing step where multiple choices exist that might influence overall classification performance. This was evaluated by comparing performance when using three different reference montages (surface laplacian reference, common average reference, and averaged mastoid reference) as well as when the referencing step was omitted. The results are presented in Figure 3D. No significant differences in overall performance were found, but classification performance was highest overall for the mastoid reference, suggesting that the signal-to-noise benefits provided by a mastoid reference that have previously been described (Kujala et al., 2007; Duncan et al., 2009) might also contribute to higher classification rates.

Two final preprocessing steps were evaluated: additional downsampling of the data, and reduction of the number of electrodes included in the final montage. These steps have important consequences for the classification problem: the reduction of the number of temporal (i.e., number of samples) and spatial (i.e., number of EEG channels) dimensions leads to overall reduction in the dimensionality of the data. As previously mentioned, dimensionality reduction implies that fewer training examples are needed to obtain optimal performance. Another benefit of removing EEG channels is the use of less electrodes during measurements, which saves time during cap-fitting.

The effects of temporal and spatial downsampling on cross-validation rates are presented in Figures 3E,F, respectively. A contrasting picture emerges. While performance significantly improves when downsampling from 256 Hz to 128 Hz [*t*_(13)_ = −2.360, *p* < 0.05], performance is reduced overall when the number of electrodes is decreased from 64 to 32 [*t*_(13)_ = 2.956, *p* < 0.05]. There are several factors that might underly this difference in spatial vs. temporal features. Firstly, in the temporal dimension, the sampling rate determines the Nyquist frequency, and thus the spectral range of activities that are captured in the data. The data has been filtered between 0.5 and 13 Hz, meaning that sample rates of 26 Hz and higher should capture the range of activity in the data. By reducing the sample rate to 32 Hz, the dimensionality of the data has been reduced by 77.5%, from 8192 dimensions to 1024. This may impact overall performance. Secondly, the ability of the classifier to optimally represent the spatial structure of both class-relevant signals and class-irrelevant noise might also depend on the relative density of electrode placement. Thus, reduction according to the 10–20 system is potentially suboptimal. Several previous studies have investigated the use of spatial filtering techniques (i.e., weighting of specific electrode locations) in the context of EEG pattern classification. Brunner and colleagues showed that the output of an infomax-based ICA could be used as a spatial filter to improve classifier performance in a dataset collected in an imagined movement paradigm (Brunner et al., 2007). Farquhar and Hill also showed that a technique known as spatial whitening could be used to attain similar improvements in performance as with ICA (Farquhar and Hill, 2013).

**Figure 4:**
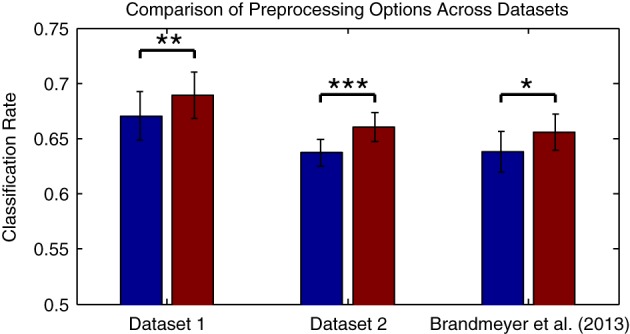
**Comparison of preprocessing effects on classification performance across datasets**. The two datasets presented in this paper, as well as data collected in Brandmeyer et al. (2013) from 11 native English speakers using English language speech stimuli, were used to evaluate whether the optimal parameters for preprocessing steps observed for Dataset 1 generalized to novel datasets and types of stimuli. Performance was compared when using the original filter (1–25 Hz band-pass) and sampling rates (256 Hz for Datasets 1 & 2, 128 Hz for Brandmeyer et al., 2013), and when using the optimal filter (0.5–13 Hz) and sample rate (32 Hz). Significant improvements in mean performance were observed for all three datasets. The significance of paired-sample *t*-test comparisons is indicated using asterisks: ^*^p < 0.05, ^**^p < 0.01, ^***^p < 0.001.

Compared to the results obtained with the original preprocessing parameters, classifier performance is significantly improved using the updated parameters [*t*_(13)_ = −4.216, *p* = 0.0001]. It's worth knowing whether the improvements obtained through changes in filter settings and by downsampling generalize to other datasets collected in an MMN paradigm and using different types of stimuli. This was evaluated with Dataset 2, as well as using a subset of the data from Brandmeyer et al. (2013) collected in an MMN paradigm from 11 native-English speakers using English phonemes. This study made use of similar initial preprocessing steps and a decoding analysis. Significant improvements in average classification rates were obtained using the current parameters for both Dataset 2 [*t*_(11)_ = −4.484, *p* < 0.001] and for the data from Brandmeyer et al. (2013) [*t*_(10)_ = −2.479, *p* < 0.05] (see Figure 4). This implies that these parameters may serve as useful guidelines for EEG based-decoding approaches using an MMN paradigm. A summary of the optimized parameters can be found in Table 2. The remainder of this article makes use of data pre-processed using these parameters.

**Table 2:** **Summary of optimized preprocessing parameters**.

**Step**	**Parameters**
Bad-channel detection and repair	Individual electrodes with offsets greater than ±35 mV or 50 Hz greater than 1000 μV^2^ repaired using spherical-spline interpolation of neighboring electrodes on a per-epoch basis
ICA-based artifact removal	Infomax-based ICA procedure used to obtain independent component transform of EEG data. Individual components with variance above overall mean variance of the dataset selected for removal, visual inspection of selected components followed by reproduction of data to EEG channels
Filter	Band-pass between 0.5 and 13 Hz
Artifact rejection	±75 μV
Rereferencing	Average of left and right mastoid leads
Resampling	32 Hz

### 2.2. Effects of collection methods and dataset size

Data collection methods and the amount of available data can also influence classifier performance. For example, ERP component amplitudes will often decrease during measurements due to habituation (Ritter et al., 1968; Fruhstorfer, 1971; Sams et al., 1984). Previous BCI research has demonstrated that such habituation effects can lead to reduced performance (Sellers and Donchin, 2006; Salvaris and Sepulveda, 2009).

**Figure 5:**
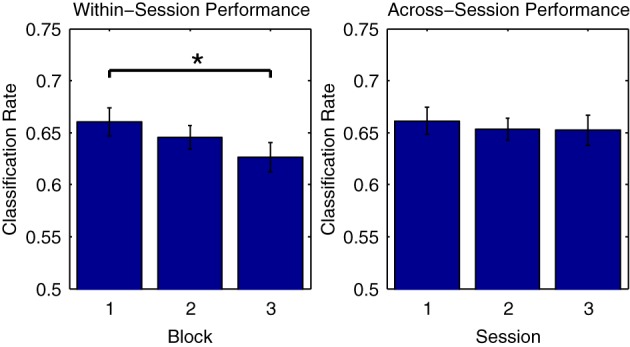
**Comparison of performance across measurement blocks and sessions**. Classification rates obtained using individual measurement blocks from Dataset 2 were compared. While no significant difference in performance was found for the initial blocks in each of the three sessions, a significant decrease in performance from the first to the third block of the first session was observed. This may be due to a number of factors, including response habituation and experimental fatigue, both of which have been shown to influence ERP classification performance (Sellers and Donchin, 2006; Salvaris and Sepulveda, 2009). Such effects should be carefully considered during the design of experiments and procedures that make use of ERP classification. The significance of paired-sample *t*-test comparisons is indicated using asterisks: ^*^p < 0.05.

Figure 5 illustrates this phenomenon using Dataset 2, for which three consecutive measurement blocks were available for each of three separate measurement sessions on different days. On the one hand, looking at average classifier performance across the three blocks of the first session, classification rates progressively decrease, with the difference in performance between the first and third blocks reaching significance [*t*_(11)_ = 2.979, *p* < 0.05]. On the other hand, when comparing the initial blocks recorded in each of the three sessions, no significant differences in the average performance rates are observed. This suggests that class-relevant differences in brain responses to standard and deviant trials are relatively enhanced in the initial portions of a given measurement session. In support of this, an analysis of the mean MMN component amplitudes across the three blocks measured in the first session showed a gradual reduction in negativity (First block: −3.90 μV, second block: −3.71 μV, third block −3.43 μV), although these differences were not significant.

Another factor that influences performance is the amount of MMN data used for classifier training. This is an issue shared with ERP paradigms that analyze individual MMN responses. It has been suggested that 200–300 deviant trials should be collected to reliably estimate individual MMN amplitudes, latencies and statistical significance(Bukard et al., 2007; Duncan et al., 2009; Bishop and Hardiman, 2010). Previous analyses looking at the effects of EEG dataset size on classification performance have also shown consistent improvement in classification accuracy as more data becomes available (Blankertz et al., 2011; Farquhar and Hill, 2013).

**Figure 6:**
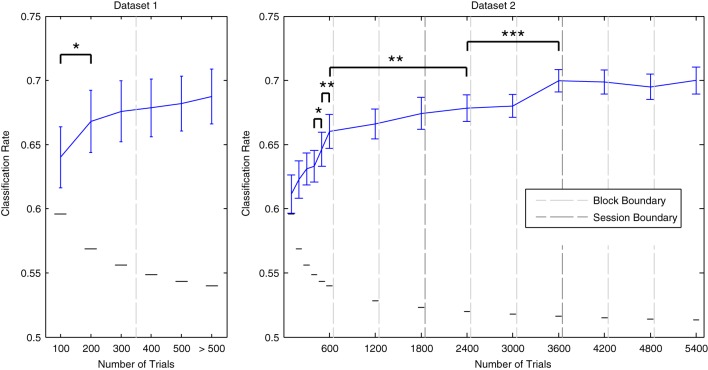
**Effects of dataset size on average cross-validation rates**. Individual data from Dataset 1 and Dataset 2 were used in incremental steps to train classifiers on increasingly larger amounts of data. The order in which the data was collected was preserved. Block/session boundaries are indicated along with binomial confidence intervals for the maximal number of available data points in each iteration. The amount actually used for some participants was slightly reduced at certain intervals due to the removal of data epochs during the artifact rejection preprocessing step. Data from additional blocks collected for some participants in Dataset 1 were utilized in the final iteration. With respect to Dataset 1, a significant increase in performance was observed for the initial increase in dataset size, from 100 trials to 200 trials. Performance was highest when using the complete dataset. For Dataset 2, data from each of the three blocks in each of the three measurement sessions were incrementally added to the analysis. Significant improvements were observed up to the inclusion of 3600 data epochs (i.e., the full data sets from the first and second sessions). From here on, average classification performance remained at approximately 70%. Error bars indicate the standard error across participants, while the small horizontal lines indicate the binomial confidence interval for the maximum number of data epochs that were available. Consecutive pairs of incrementation steps for which significant improvements in performance were observed are indicated with brackets and asterisks: ^*^p < 0.05, ^**^p < 0.01, ^***^p < 0.001.

The effects of incrementally increasing dataset size on cross-validation performance are illustrated in Figure 6. For both datasets, performance improves as more data are included. Gains in performance are greatest during the initial increases in dataset size. For Dataset 1, performance significantly improved when the amount of total trials was increased from 100 to 200 [*t*_(13)_ = −2.515, *p* < 0.05]. For Dataset 2, significant increases were observed when increasing from 400 to 500 [*t*_(11)_ = − 2.337, *p* < 0.05], and from 500 to 600 trials [*t*_(11)_ = −3.448, *p* < 0.01]. As Dataset 2 contained a much larger number of total data epochs (up to 5400 per individual participant), it was possible to examine whether these gains continue when including data recorded across multiple sessions. Additional performance benefits were observed when increasing the amount of data utilized from 600 to 2400 trials [*t*_(11)_ = −3.176, *p* < 0.01], and from 2400 to 3600 trials [*t*_(11)_ = −6.675, *p* < 0.001]. Indeed, performance was maximal (70% on average) when utilizing all data from the first two sessions or from all three sessions combined. We conclude that decoding analyses of MMN data from multiple measurement sessions can benefit from pooling data within and across sessions. This benefit seems to outweigh the decrease in performance observed across consecutive blocks measured within a session.

### 2.3. Evaluation of class-relevant features of MMN data

We now illustrate methods for evaluating specific class-relevant features of these data. In general, when applying classification methods to time-domain EEG data, it is useful to understand which specific ERP components contribute to classifier performance (Blankertz et al., 2011). While we would obviously expect the MMN component to contribute to classifier performance in these analyses, it is not the only component of the auditory ERP modulated during the presentation of a deviant trial. For instance, Figure 1 illustrates that the P3a response is clearly elicited by deviant trials. We illustrate two methods for evaluating these class-relevant features using Dataset 1: so-called “searchlight” methods (Haynes and Rees, 2006; Blankertz et al., 2011; Chen et al., 2011; Herrmann et al., 2012) in the temporal and spatial domains, and area-under-the receiver operating characteristic, or area-under-the-curve (AUC) scores (Fawcett, 2006).

Individual components such as the MMN are defined by both the time intervals in which they occur as well as by the spatial distribution of the corresponding scalp potentials. One method for evaluating the contribution of individual time-points and EEG channels is to perform a searchlight analysis in the temporal and spatial domains. When a searchlight analysis is performed in the spatial domain, a series of classification analyses are performed at each individual EEG channel using all available time-points. Channels at which the various ERP components distinguishing the two types of trials are maximal will show the highest cross-validation rates. Conversely, in the temporal domain, a series of classification analyses are performed on each available time-point using all available EEG channels. Time-points with large differences in the amplitudes of the scalp potentials across channels for the two types of trials will generally show the best performance.

**Figure 7:**
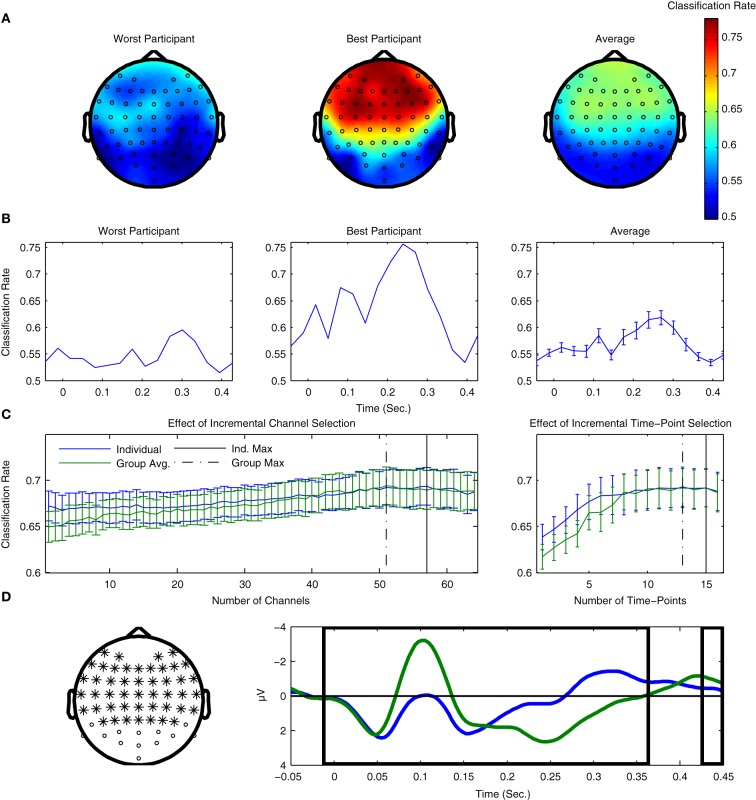
**Use of searchlight procedures for spatial and temporal classification of Dataset 1. (A)** Classifier performance using individual EEG channels. Cross-validation rates at individual EEG channels are shown for the two participants with the lowest and highest cross-validation rates, as well as the average rates across all participants. The fronto-central electrode locations typically associated with maximal MMN responses are where the highest performance was observed. **(B)** Classification performance using individual time-points. Performance for the same two participants and the average across participants are shown. Peaks in performance can be seen for both the MMN and P3a component time-ranges, as well as an additional peak near stimulus onset. When comparing the timing of the peaks for the two individual participants, a relative shift in their latencies can be observed. Similar shifts in ERP component latencies are typically associated with individual differences in perceptual discrimination and task performance. **(C)** Effects of incremental inclusion of channels and time-points based on searchlight performance results. Cross-validation performance rates were evaluated using an incremental procedure in which either individual EEG channels or time-points were included on the basis of their performance ranking in the previous searchlight analyses. This was done using the rankings obtained for both individual participants as well as the average ranking across participants. The peak performance is indicated for both individual and group rankings. **(D)** Selection of optimal channels and time-points on the basis of average rankings. 51 EEG-channels and 13 time-points are highlighted on the basis of the maximal average cross-validation rates obtained using the incremental procedure. The selection of optimal EEG channels can potentially reduce the size of the cap-montage used during measurements, thus reducing the time needed for cap-fitting by approximately 20%, as well as further reducing the dimensionality of the data used during classifier training (from 64 channels × 16 time points = 1024 dimensions to 663).

The results of both types of searchlight analyses are presented for two individual participants as well as averaged across all 14 participants from Dataset 1 in Figure 7. Figure 7A presents results in the spatial domain. Here, the fronto-central channels typically associated with MMN peak amplitude clearly show the highest classification performance, indicating that these channels provide the most information regarding the two classes of ERPs. Conversely, occipital channels show relatively poor performance, which is not surprising, given what is known about the scalp distributions associated with auditory evoked potentials (Bukard et al., 2007).

Figure 7B plots the equivalent results for the searchlight analysis performed in the temporal domain. Here, clear peaks in both the individual and average data can be seen for time-points corresponding to the MMN and P3a intervals, and, to a lesser-extent, at time-intervals immediately preceding and following stimulus onset. Interestingly, classifier performance of individual time-points is maximal within the time-interval associated with the P3a interval, which suggests that the attentional shift associated with the P3a component corresponds to single-trial brain responses that are relatively robust compared to the MMN component.

In the section on preprocessing, it was shown that temporal downsampling led to an overall improvement in performance while spatial downsampling had a negative impact on performance. The results of both searchlight procedures can also be used to investigate the effects of gradually increasing the number of channels and time-points utilized in the classification analysis. By sorting these channels and time-points on the basis of both individual and averaged results, an incremental procedure can be used to estimate which subsets lead to optimal classifier performance. We prefer this incremental procedure to a more comprehensive evaluation of electrode combinations due to the very large (approximately 10^89^) number of possible permutations.

These results are presented in Figure 7C. Analyses based on both individual and average searchlight results converge at relatively similar points: between 7 and 13 channels and 1–3 time-points can be removed without reducing overall performance. Results based on the average searchlight results across participants lead to a slightly smaller number of channels and time-points than the individual results. These channels and time-points are illustrated in Figure 7D. In particular, the results of channel removal could potentially be used to reduce the number of electrodes in the optimal cap-montage to 51, a reduction of approximately 20% that could positively impact cap-fitting times in online settings. Further reductions appear to negatively impact performance. It has been suggested that the use of a larger number of EEG channels generally improves the performance of ERP component classification, provided that appropriate regularization methods are used (Blankertz et al., 2010).

Methods also exist for evaluating individual spatio-temporal features of the data. An AUC analysis represents one such approach that has proven useful in the context of classification analysis (Fawcett, 2006). AUC scores are derived from the receiver operating characteristic (also referred to as the ROC curve), which is an analytical tool developed in the context of signal detection theory, and which is also widely used in psychophysics (Green and Swets, 1966). Essentially, the AUC score (between 0 and 1) quantifies the ratio of true-positives to false-positives in a signal detection task. This has proven useful in the context of machine learning and pattern classification, as it provides a richer measure of classifier performance than accuracy alone.

**Figure 8:**
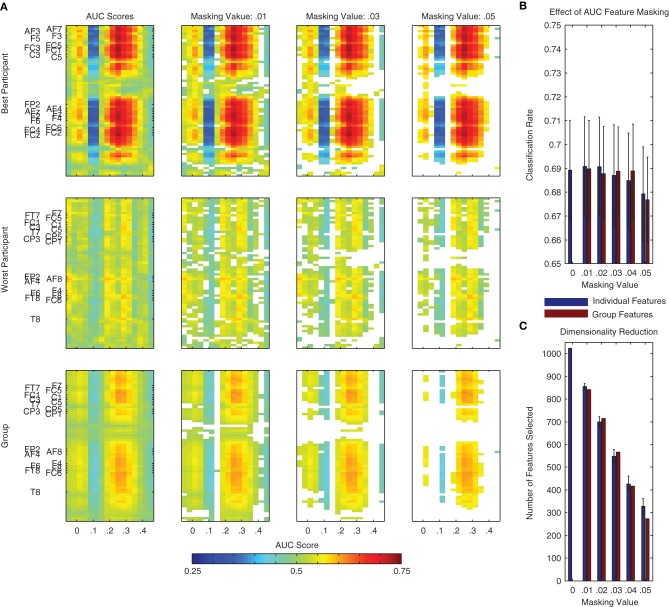
**Area under the receiver operating curve (AUC) scores and masking procedure**. AUC scores are often used to evaluate the class-relevant information present in datasets used for classifier training. Additionally, they can be used to select the most relevant dimensions of the data during classification analysis. **(A)** Such a procedure is illustrated here using the same two individual participants as in Figure 7 as well as using all participant data from Dataset 1. Individual dimensions were selectively masked (i.e., removed from the dataset) on the basis of their AUC score. This masking value was defined as |*AUC* − 0.5|, that is, the absolute value of the AUC Score minus 0.5. This is effectively a measure of a feature's distance from the no discrimination line of the ROC plot. Additionally, we performed this analysis on the basis of AUC scores calculated using both the individual and the group data. **(B,C)** Using features selected on the basis of the group data and a masking value of 0.04, it is possible to reduce the dimensionality of the data by over half (from 1024 to approximately 400, shown in panel **C**) without a reduction in average performance (see panel **B**). The remaining dimensions correspond approximately to the two clusters observed in the statistical analysis presented in Figure 1 (i.e., MMN and P3a components) as well as to additional activity around stimulus onset and between 400 and 450 ms following stimulus onset.

An AUC score of 0.5 indicates chance level performance, and suggests no discriminative information is available. In the context of the present binary classification problem, we would like to know if a given spatio-temporal dimension of the data provides information about the two classes (i.e., standard and deviant trials). Following an analysis of each individual dimension, the scores can be visualized as a two-dimensional image (i.e., space x time), similar to that used for visualizing the grand-average difference wave in Figure 1. Examples of these plots can be found in Figure 8A for the same two individual participants as in Figure 7, as well as for the entire dataset. Features corresponding to the MMN and P3a components can be seen at the expected channels and time-points.

Similarly to the incremental procedure used to select channels and time-points on the basis of the searchlight analysis, the AUC scores for individual spatio-temporal dimensions can be used to incrementally reduce the overall number of dimensions in the data used to train a classifier. This procedure is illustrated using both individual and group data in Figure 8B. Corresponding effects on classifier performance and the data dimensionality are presented in Figure 8C. Similarly to the searchlight procedure, the results of this analysis suggest that specific channels and time-points can be removed from the data without affecting overall performance.

While searchlight and AUC methods both offer a means of evaluating features that contribute to classifier performance, their use in selecting features for removal does not lead to any substantial gains in performance relative to the performance obtained following the optimization of preprocessing parameters. This is due to the fact that both methods are essentially univariate in nature, while the classifier training methods are multivariate. In essence, they fail to capture specific patterns in the covariance of multiple features useful for distinguishing class-relevant signals mixed with class-irrelevant noise. In contrast, the gradient descent and regularization methods employed during classifier training to determine the optimal weighting of individual features have in fact already selected the most informative features of the data. However, both methods provide tools for understanding which features of the data contribute to classification performance.

One final method of selecting features for classification is based on the cognitive mechanisms underlying the generation of specific ERP components. In the case of auditory mismatch data, two principal components distinguish the standard and deviant trials: the MMN and the P3a. While the MMN reflects pre-attentive sensory processes that occur in bilateral auditory cortex, the P3a component reflects subsequent activity generated in frontal cortex that is associated with an attentional switching mechanism (Näätänen et al., 2007; Polich, 2007). While these processes are essentially intertwined, they reflect two distinct forms of brain activity related to perception and attention. In the context of different perceptual tracking and neurofeedback applications, it may be the case that the decoding analysis should focus on one or the other component, depending on which specific cognitive processes are deemed to be most relevant.

**Figure 9:**
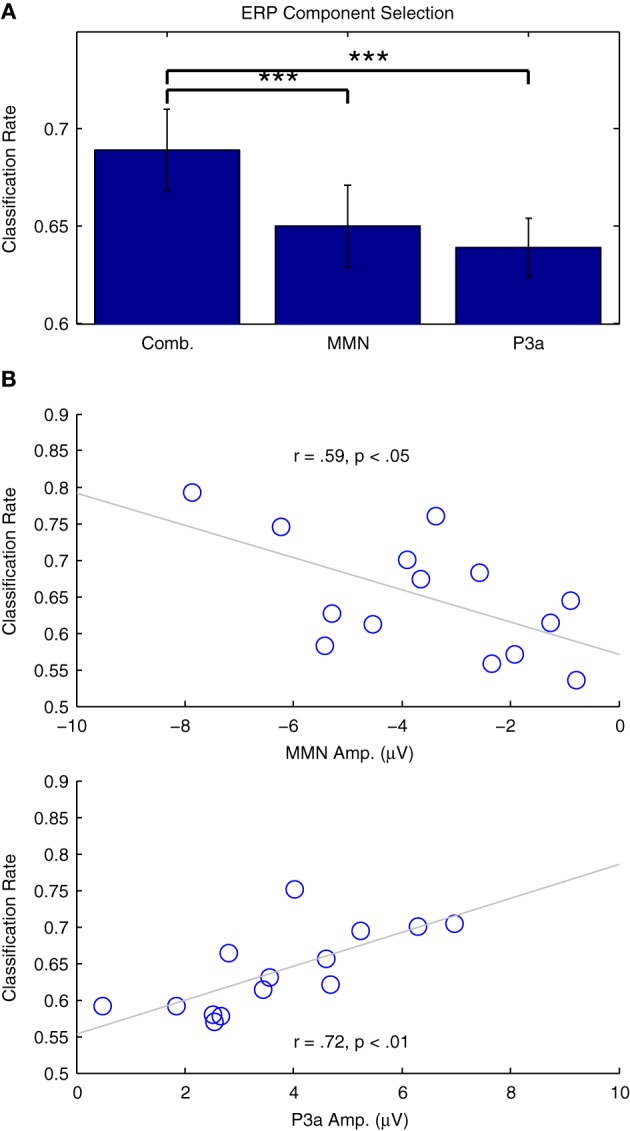
**Classification of individual ERP components. (A)** Two classification analyses were performed using individual participant's data from Dataset 1. Subsets of the data from the time windows associated with the MMN (82–207 ms) and P3a (238–363 ms) were selected and classified. Relative to analyses using the entire time range (−50 to 450 ms), cross-validation rates in both of these analyses were significantly lower, indicated using asterisks: (^***^p < 0.001 **B**) Correlation analyses of the individual results with the ERP component amplitudes measured using the individual difference waves were significant for both the MMN and P3a, indicating that classifier performance is strongly related to the amplitude of these components.

Figure 9A presents the results of two additional classification analyses performed using only data in the time intervals associated with the MMN and P3a responses, relative to the performance obtained using the entire 500 ms window (−50 to 450 ms) previously established. Classifier performance significantly decreased for both the MMN [*t*_(13)_ = 4.715, *p* < 0.001] and the P3a [*t*_(13)_ = 4.796, *p* < 0.001] time windows. This is not surprising, given that both windows provide information about the types of trials being observed, and thus offer some redundancy with respect to the binary classification problem. Additionally, the relationship between classifier performance and individual ERP component amplitudes is presented in Figure 9B. In both cases, strong correlations suggest that the analyses successfully capture relevant single-trial features in the data. These results pose a trade-off with respect to the specificity of a particular classification analysis (i.e., the focus on specific cognitive processes and associated ERP components) and the overall performance that can be expected from a given analysis.

The three approaches used to evaluate class-relevant features in auditory mismatch data all tell a similar story: single-trial brain responses measured during standard and deviant trials are primarily distinguished by activity in time-windows corresponding to the MMN and P3a ERP components, with maximal differences at fronto-central electrodes. Additional brain activation in early (0–50 ms) and late (400 and 450 ms) time windows relative to stimulus onset are also informative. In essence, the classifiers trained in the present analyses make use of data features corresponding to a sequence of perceptual and cognitive processes triggered by a stimulus. Single-trial EEG measurements are modulated in a consistent manner during deviant trials, providing the classifier with features that can be used to predict which type of trial has been observed.

### 2.4. Estimating generalization performance

While the previous cross-validation methods are useful for estimating performance in the context of a particular classification problem, the ability to generalize to new datasets lies at the heart of real-world systems that use classifiers for different applications. This is referred to as “generalization” or “transfer learning” (van Gerven et al., 2009). Typically, generalization performance is lower than cross-validation performance due to non-stationary features that aren't adequately represented in the training data. In EEG data, many factors influencing the measurement of class-relevant brain signals will change between the collection of training data and the subsequent use of the trained classifier, such as the quality of electrode connectivity and the individual's subjective state (i.e., fatigue, concentration) (McFarland and Wolpaw, 2005; Blankertz et al., 2010). These effects are more pronounced in paradigms that make use of across-session designs in which training data is collected on one day, followed by online use of the classifier on subsequent days. Therefore, it is useful to estimate performance in these settings.

**Figure 10:**
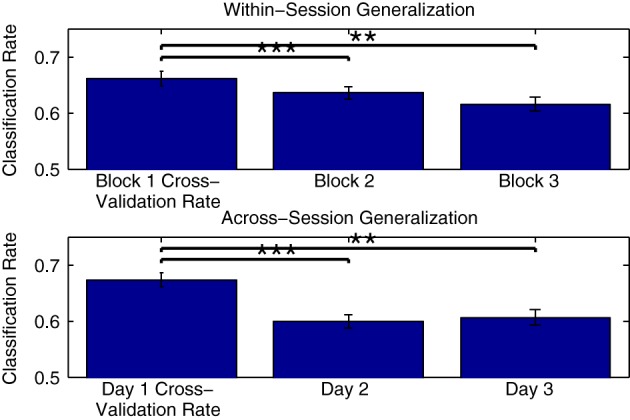
**Within-participant generalization across measurement blocks and sessions**. The ability of classifiers trained on individual data to generalize to novel data collected during either the same or in different measurement sessions was evaluated using Dataset 2. In the within-session analysis, classifiers were trained using data collected in the first measurement block and then tested using data from the second and third blocks. In the across-session analysis, classifiers were trained using data from all three blocks measured in the first session, and tested using the initial blocks measured in the second and third sessions. For both analyses, a significant drop in performance was observed relative to the cross-validation rates obtained when training the classifiers. The significance of these paired-sample *t*-test comparisons is indicated using asterisks: ^**^p < 0.01, ^***^p < 0.001. The drop across sessions was more severe, and could be potentially due to a number of factors, including changes related to the placement of electrodes as well as differences in the subjective states of the individual participants on different days.

Figure 10 shows the mean generalization performance obtained for individual participants using Dataset 2, for both within- and across-session designs. For the within-session data, performance decreases significantly in the second [*t*_(11)_ = 4.569, *p* < 0.001] and third measurement blocks [*t*_(11)_ = 4.222, *p* = 0.0014] relative to the cross-validation rates obtained when training a classifier using data from the first block, following a similar pattern as seen with the cross-validation rates obtained when training classifiers using these two blocks (shown in Figure 5). The mean across-session generalization performance was also significantly lower for the second [*t*_(11)_ = 4.340, *p* = 0.0012] and third [*t*_(11)_ = 5.391, *p* < 0.001] sessions than the cross-validation rates obtained when training a classifier using all available data from measurements on the first session. Here, the drop in performance is larger, given that the initial cross-validation rates are also higher than when training using only data from the first measurement block. However, in both the within- and across-session versions of the analysis, the average generalization rates are still significantly above chance level, with performance approximately between 60 and 65%.

Another form of generalization that has drawn interest is the ability of classifiers to generalize across participants. Here, a classifier is trained using data from multiple individuals, and is then tested using another individual's previously unseen data. Such analyses have been used to investigate the extent to which various forms of brain activity associated with auditory perception and language processing overlap between individuals (Schaefer et al., 2011; Herrmann et al., 2012; Brandmeyer et al., 2013). The use of cross-participant classifiers has several potential advantages: firstly, the use of data from multiple participants provides a means for assembling relatively large datasets for classifier training, which, as was discussed previously, can improve performance. Additionally, the use of a previously trained cross-participant classifier could eliminate the need for collecting training data during online applications, thereby reducing the amount of time required along with associated issues such as response habituation and fatigue. On the other hand, cross-participant classifiers might also fail to capture individual variability in class relevant features.

**Figure 11:**
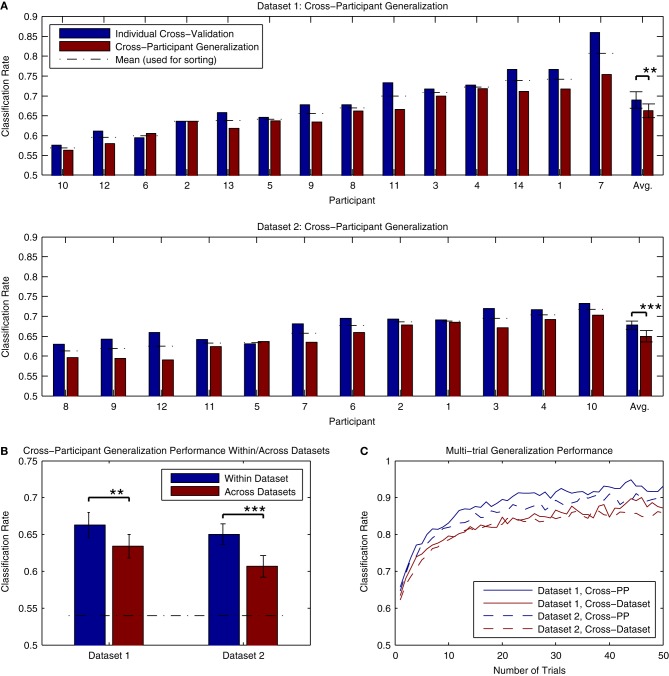
**Classifier generalization to novel participants and datasets. (A)** Both datasets were used to perform a cross-participant analysis of generalization performance, in which a series of classifiers were trained using all but one participant's data, and then tested on the remaining participant. The results of these analyses are presented along with the individual cross-validation rates for comparison. For both datasets, a significant decrease in performance relative to the individual cross-validation rates was observed (approximately. 2–3% reduction). This drop in performance is most likely due to the variability of ERP features across individual participants, such as component latencies and spatial topographies. However, it also appears that these features generalize across participants to the extent that average classifier performance of 65% or higher is still possible. **(B)** The ability to generalize across datasets was evaluated by training a classifier using all available data from one of the datasets, and testing it using the data from the other dataset. The average performance of these tests across individual participants is shown relative to the cross-participant classification rates obtained when training and testing within each dataset. As expected, a significant drop in performance was observed in both cases. However, in both cases, average classification rates were above 60%, well above the binomial confidence interval, and higher than the performance observed for within-participant generalization across sessions. **(C)** Classifier predictions can be combined across multiple trials to increase prediction accuracy. This was evaluated for both datasets using the results of the within-dataset cross-participant analysis and the across-dataset analysis. Results shown are averaged across all participants within each dataset. For panels **(B,C)**, the significance of the statistical comparisons are indicated using asterisks: ^**^p < 0.01, ^***^p < 0.001.

The results of cross-participant analyses of both Dataset 1 and Dataset 2 are presented in Figure 11A. Here, we present a comparison of the cross-validation rates obtained when training a classifier using individual data and the performance obtained when applying a classifier trained using data from all other participants (within the same dataset) to the same individual data. The analysis for Dataset 1 made use of all available data, while the analysis of Dataset 2 made use of 3 blocks of data (maximally 1800 trials per participant). On average, there was approximately a 2–3% decrease in performance relative to the individual cross-validation rates. This decrease was significant for both Dataset 1 [*t*_(13)_ = 3.876, *p* < 0.01] and Dataset 2 [*t*_(11)_ = 5.056, *p* < 0.001]. This decrease is substantially less than the across-session generalization performance that was seen in the analysis of individual data from Dataset 2, suggesting that cross-participant classifiers might offer some advantages in paradigms making use of longitudinal measurements. Classification rates for all participants in both datasets were significantly above chance-level, based on the binomial confidence intervals. In order to verify that that these results were not influenced by a bias in the data, we also conducted a permutation test of the results by shuffling the labels of the individual epochs (10000 permutations) and estimating the *p*-value as the fraction of the permutation distribution with scores greater than or equal to the observed classification rate. Here, all of the results were highly significant (*p* < 0.001, except for PP10 in Dataset 1, *p* = 0.0025).

An additional question regards the extent to which these classifiers generalize to novel datasets collected using different stimuli and measurement paradigms. This was evaluated by applying the cross-participant classifiers trained in the previous analysis (using all participants) to the other dataset. The results can be seen in Figure 11B. While significant decreases in performance relative to the cross-participant generalization rates were observed for both Dataset 1 [*t*_(13)_ = 3.341, *p* < 0.01] and Dataset 2 [*t*_(11)_ = 4.735, *p* < 0.001], performance for both datasets was on average higher than the across-day generalization performance obtained when using individually trained classifiers. Only one participant in Dataset 1 (PP10, classification rate = 0.521) did not show classification rates significantly above chance level; classification rates for the remaining Dataset 1 participants (range: 0.566–0.734) and all Dataset 2 participants (range: 0.541–0.690) were significantly above chance. Such results suggest that the brain responses collected using different variations of the MMN paradigm and stimuli generalize well enough to obtain reasonably high classification accuracies. This in turn opens the door for online paradigms that make use of variable stimuli and sequence types, depending on required performance.

The average classification rates in the preceding analyses of generalization performance fall approximately between 60 and 66%. In BCI paradigms that make use of single-trial classification, it is common to combine predictions made for multiple data epochs in order to increase the accuracy of the predictions. Recalling equation 1, classifier predictions can be combined by summing the decision values obtained for each epoch *x*_*i*_ in a set of *k* epochs.

Figure 11C presents an analysis of the improvements in accuracy obtained when combining single-trial predictions across differing numbers of trials. The analysis made use of the predictions obtained in the cross-participant and cross-dataset analyses of generalization performance. As can be seen, average classification rates rapidly improve when combining up to 10 trials, with the rate of improvement gradually decreasing thereafter. While the combination of multiple trials will lead to an increase in the accuracy of predictions, it comes with a trade-off: collecting multiple epochs of data requires additional time, and thus decreases the speed at which the system is able to make predictions. In the BCI literature, the term “bit rate” is used to describe the informational output of a system (van Gerven et al., 2009), and is a function of the classification accuracy and the time require to obtain the data. Lower single-trial classification rates will lead to systems with a lower bit rate, due to either the number of incorrect predictions made or the increased amounts of time needed to collect multiple epochs in order to increase the accuracy of the predictions. The role of this trade-off between time and accuracy in the decoding of MMN responses for different applications is discussed in the subsequent sections.

In summary, we have shown that classifiers trained on individual and group MMN data generalize well to new datasets. While performance drops relative to the cross-validation rates obtained during classifier training, generalization performance within- and across-sessions, as well as across participants and data sets, is on average in the range of 60 to 65%, and can be improved by combining classifier predictions across multiple, non-overlapping trials.

## 3. Online tracking of perceptual discrimination

**Figure 12:**
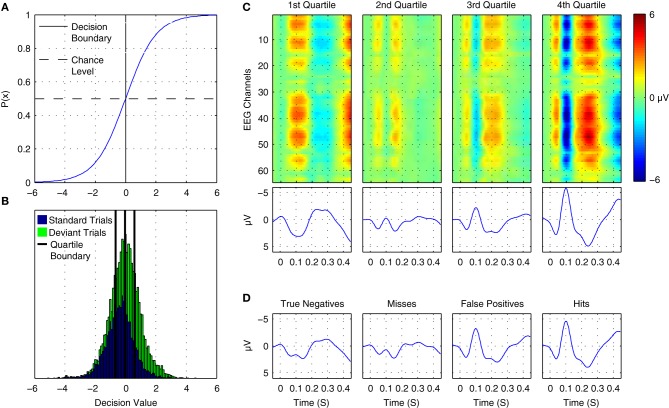
**Online tracking of single trial brain activity in an auditory mismatch paradigm. (A)** The logistic regression function. **(B)** Sorted single-trial classifier decisions for test set data in the cross-participant analysis of Dataset 1 (see Figure 11A, upper panel). A division of the trials into quartiles on the basis of the decision values is shown using thick black lines. **(C)** Grand average ERPs for sorted single-trial data in each quartile. Upper panels show ERP activations at all 64 EEG channels, while the lower panels show the averaged waveforms obtained at nine fronto-central electrodes (see Figure 1 for reference). A clear shift in both the number and amplitude of the components in the obtained ERPs is visible across the four subsets of the data. **(D)** Grand average ERPs based on classifier signal detection performance. Correct and incorrect decisions for both the target (deviant) and non-target (standard) classes are used to group trials. As can be seen, shifts in the relative amplitudes of multiple ERP components are associated with correct and incorrect decisions for both standard and deviant trials.

The previous sections illustrated how classification analyses can be used together with single-trial data collected in an MMN paradigm. We now illustrate how these methods can be used to track the brain activity underlying the MMN and P3a responses online. In particular, we show how the output of a logistic regression classifier can be interpreted as a probability that a particular type of brain activity (i.e., the MMN response) has been observed in a given epoch of data. Logistic regression classifiers make use of the logistic function, which always takes on values in the range [0, 1]:

(3) $$p(+|x)=\frac{1}{1-e^{-f(x)}}$$

where *p*(+|*x*) is the posterior probability of the positive class given a data epoch *x*. A plot of the logistic function is shown in Figure 12A. Thus, the output can be interpreted as the posterior probability that the positive class has been observed by providing it as input to the logistic function. The probability of the negative class is equivalently 1 − *p*(+|*x*). Differences in the observed probabilities obtained when applying a classifier to a given data epoch reflect differences in the class-relevant features of the data. Thus, the observed probabilities can be used to order or index data on the basis of these features.

Figure 12B presents the distribution of decision values obtained for cross-validation test-set data in Dataset 1 during the cross-participant generalization analysis. Standard and deviant trials are plotted with separate colors. The corresponding probabilities for the trials can be inferred by referencing the logistic function plot above it. By dividing this data into subsets on the basis of ordered classifier decisions, it is possible to track underlying changes in the single-trial ERP morphology. Figure 12C displays ERP images and ERP waveforms for 4 subsets of the data. No distinction between standard and deviant trials or participants is made. Rather, data are grouped on the basis of classifier decisions. Each subset of the data has a distinct morphology, with the primary differences related to the N1/MMN components, the P2/P3a components, and a late component between 400 and 450 ms following stimulus onset. For example, when comparing averaged data from the first quartile and the fourth quartile, the opposite patterns of activity can be seen at approximately 100, 250, and 425 ms. Moreover, the gradual change in the average amplitude of the corresponding ERP components can be clearly observed in the second and third quartiles. This indicates that the decision values are tracking amplitude fluctuations and spatial shifts in brain activity at these specific points in time in the single-trial data. An alternative grouping of trials is presented in Figure 12D. Here, grand average ERPs are presented based on whether a trial was classified correctly. This corresponds to “Hits” and “Misses” for deviant trials, and to “True Negatives” and “False Positives” for standard trials. A similar pattern to the ERPs in Figure 12C emerges, suggesting that the trial-to-trial fluctuations in brain responses at specific time points within a given trial-type are being tracked by the classifier.

The ability to track fluctuations in specific ERP components at the single-trial level offers the possibility to investigate the short-term dynamics of the brain activity underlying their generation. The assumptions made by this approach are essentially the same as those upon which general ERP methodology is based. In essence, the time-locked brain activities measured in single-trial EEG data reflect meaningful sources of variation, such as individual differences (e.g., expert/non-expert), task differences (e.g., target/non-target trials), habituation effects or stimulus differences (e.g., standard/deviant trial). Using averaging, these differences in the time-locked ERPs are typically visible to the eye, and can be subjected to statistical analysis. However, such methods have no means of investigating trial-to-trial fluctuations in the generation of these response. Methods such as ERP images (Makeig et al., 2004) have been developed as a means of sorting data from individually measured trials post hoc using a variable of interest, such as oscillatory phase. Recently, models of MMN generation based on the free-energy principle have been used to explain trial-to-trial variation in MMN amplitudes (Lieder et al., 2013). The continuous output of a classifier trained on representative data provides another means of indexing single-trial brain responses that can also be used online.

**Figure 13:**
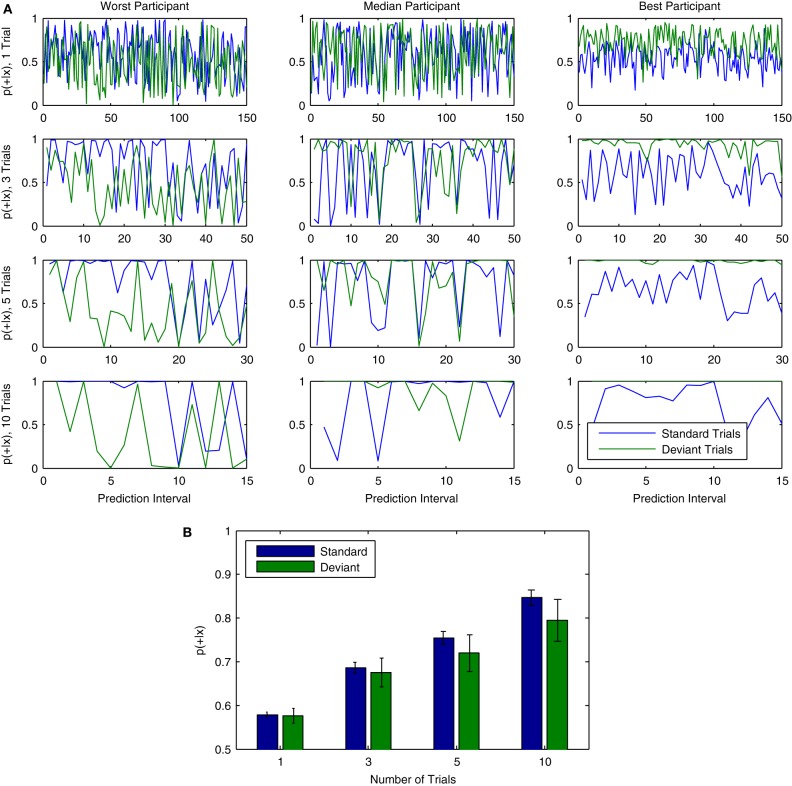
**Probabilistic interpretation of classifier output over trials. (A)** Data from three representative participants in Dataset 1 (left to right) illustrates a probabilistic interpretation of classifier decisions for single and varying group lengths of (non-overlapping) trials. Results are taken from the first 150 consecutive trials analyzed in the cross-participant analysis. From top to bottom, the number of trials used to make predictions about ongoing brain responses is varied, using either 1, 3, 5, or 10 trials. This effectively reduces the number of predictions that can be made: while 150 individual predictions are made when using only 1 trial, only 15 predictions are made when using 10 trials. As can be seen, the output becomes increasingly stable as the number of trials is increased. **(B)** Similarly to the increase in single-trial classification rates observed when combining across multiple trials (see Figure 11C), the mean of the continuous classifier output for all 150 trials increases as predictions are combined across groups of trials of increasing size.

Examples of classifier output across time can be found in Figure 13A. The results of the cross-participant analysis for 150 consecutive trials are used to visualize fluctuations in the continuous output of the classifier for both standard and deviant trials. Results are shown for three representative participants from Dataset 1. The effects of using different numbers of trials are also presented. Combining multiple trials leads to output which is generally more stable across consecutive predictions, but also reduces the frequency at which predictions are made about the data. Individual participants also show differences with respect to the relative strength of the predictions being made about standard and deviant trials, as well as the specific points in time when fluctuations in classifier output are observed. These differences are most clearly observable when combining across 10 trials.

Mean output across all participants in Dataset 1 is shown for different numbers of trials (1, 3, 5, or 10) in Figure 13B. In general, overall predictive confidence improves through the combination of multiple single-trial predictions. Continuous classifier output also tends toward higher values for standard trials than for deviant trials. This corresponds to a slightly higher single-trial classification accuracy for standard trials (67.6%, see Figure 12D “True Negatives”) than deviant trials (64.9%, see Figure 12D, “Hits”), as well as to a median decision value (see Figure 12) that is slightly negative. While these differences are small, they suggest that classifier predictions about standard trials are more reliable and confident than those about deviant trials. In the context of the auditory mismatch paradigm being investigated, one possible explanation is that there is less variation in the brain responses measured on standard trials than in deviant trials, given the greater frequency (and predictability) of standard trials. In other words, brain responses measured in standard trials may be more stable as compared to deviant trials.

Given the average single-trial classification accuracies (approximately 66%) obtained in the present analysis, one factor that will influence classifier output is change in the EEG signal unrelated to the auditory evoked potentials being analyzed. This implies that the output of the classifier is noisy. While data can be combined across multiple trials to reduce the effects of this noise, this comes with a trade-off: rather than estimating the probability of a particular set of brain responses at the single-trial level, an estimation of these responses is made across a longer period of time. This means that changes in the brain responses across shorter intervals will be mixed together, and that a delay is introduced into the tracking procedure.

The present approach of tracking brain responses underlying perceptual discrimination is in many ways similar to BCI paradigms which also make use of single-trial pattern classification analysis. A key aspect is the interpretation of classifier output as a continuous probability rather than as a binary decision. These values correspond to graded modulations of brain activity measured using EEG at time points where ERP components such as the MMN and P3a are typically observed, and as such, serve as an index of ongoing perceptual discrimination. As such, ongoing fluctuations in classifier output effectively track changes in perception online. Differing degrees of single-trial classification accuracy for standard and deviant trials are also reflected in the continuous interpretation of classifier output, suggesting that the patterns of brain activity measured in individual standard trials are somewhat more stable than in deviant trials. While the accuracy of this tracking procedure is also influenced by noise sources in the EEG signal, predictions can be combined across multiple trials in order to obtain a more stable measure of perceptual discrimination across longer time intervals.

## 4. Potential domains of application

This article has thus far presented a method for utilizing pattern classification methods and single-trial EEG data recorded in two variants of the auditory mismatch paradigm to track brain activity associated with perceptual discrimination processes. It serves as a “recipe” for online applications that aim to monitor changes in perceptual discrimination, including the effects of learning.

### 4.1. Cognitive monitoring and passive BCI

One domain where these decoding methods can be applied is cognitive monitoring, in which real-time measurements are used by clinicians and researchers to infer the cognitive state of a user. For example, the working memory load of an individual can be monitored as they engage in challenging tasks using single-trial decoding of EEG data (Brouwer et al., 2012). The ability to detect ongoing changes in working memory and cognitive load has also been proposed for use in the enhancement of aspects of human-computer interfaces (Grimes et al., 2008).

Variations in MMN response characteristics are associated with both differences in perceptual discrimination abilities and with various clinical and medical conditions. The ability to decode MMN responses at the single trial using existing classifiers and to perform a probabilistic assessment of the MMN response using a reduced number of trials and/or electrode recording channels might offer a valuable alternative in settings where assessment of brain responses augments or is preferred to behavioral assessments, but in which time is limited. This might include forms of objective audiometry (Bukard et al., 2007), which measure perceptual thresholds using auditory evoked potentials instead of behavior.

Closely related to cognitive monitoring are passive BCIs (Zander and Kothe, 2011), which measure ongoing brain responses as an auxiliary input to an interface. For instance, a computer could automatically adapt the difficulty of a given learning task on the basis of ongoing measurements of working memory load. Similarly, a passive BCI based on an auditory mismatch paradigm would be able to adapt the difficulty of an auditory learning task on the basis of a probabilistic assessment that an MMN response had been observed in the preceding trial(s). Additional work should assess how adapting the difficulty of a listening task (i.e., stimulus contrast size) influences classifier performance, as changes in contrast salience are known to influence the timing and amplitude of the MMN component (Näätänen et al., 2007).

### 4.2. Neurofeedback

Neurofeedback is an approach in which measurements of an individual's ongoing brain activity are mapped onto a feedback signal of some kind, typically in the auditory or visual modality (Hammond, 2011). The goal of neurofeedback is to modulate the targeted forms of brain activity, which are associated with particular cognitive or mental states, such as attention, concentration or anxiety (Lansbergen et al., 2011; Zotev et al., 2011). For instance, many EEG-based approaches use measurements in the frequency domain of oscillatory activity at specific electrode locations in the alpha, theta and other bandwidths (Hammond, 2011). Neurofeedback paradigms using fMRI measurements can measure activity in specific brain regions, such as the limbic system (Zotev et al., 2011) or perceptual cortices (Yoo et al., 2006; Scharnowski et al., 2012). Neurofeedback approaches have shown promise in treating various clinical disorders such as chronic pain (de Charms, 2008) and tinnitus (Weisz et al., 2011), and can also improve cognitive performance (Zoefel et al., 2011) and emotional regulation abilities (Johnston et al., 2010).

Two studies recently demonstrated that neurofeedback training using fMRI measurements of activity in early visual cortex can induce perceptual learning effects (Shibata et al., 2011; Scharnowski et al., 2012). These findings are relevant for the present method, as modulations of the MMN response have also been linked to perceptual learning (Tremblay et al., 1997; Menning et al., 2000). In particular, Shibata et al. (2011) made use of neurofeedback based on the decoding of specific activity patterns in visual cortex corresponding to three Gabor stimuli with different orientations. This was done using a logistic regression classifier. Similarly to the present method, the neurofeedback was based on a probabilistic interpretation of a classifier's output when applied to novel data during the neurofeedback sessions. Their results indicated that the mean probability that the targeted class of brain response had been observed (i.e., the feedback signal) increased on average across the course of the training sessions. This suggests that the continuous changes in classifier output observed online and provided as feedback reflect meaningful variations in the decoded brain activations, and that modulation of this feedback signal can lead to concomitant perceptual learning effects.

Given the link between MMN response characteristics and the perceptual skills underlying language and music proficiency, neurofeedback based on single-trial decoding of MMN responses has the potential to augment language and music training paradigms. Future research into MMN-based neurofeedback paradigms should investigate the specific task and stimulus parameters that optimize the effectiveness of the neurofeedback training paradigm. This might include issues such as stimulus contrast size, task instructions (active or passive use of the neurofeedback), and the number of trials used to generate feedback. The trade-off between accuracy (i.e., single-trial classification rates) and time (i.e., number of trials used to make predictions) is an issue that has been considered in the context of reinforcement learning (Cardinal, 2006). As both temporal delays and noise influence the effectiveness of a reinforcer stimulus (in this case, the classifier decisions mapped onto a feedback signal), either the use of single- (noisy) or multi-trial (delayed) classifier output will reduce the effectiveness of the feedback. It is worth noting that the mean classifier output in the study of Shibata et al. (2011), which was used to induce perceptual learning via neurofeedback training, was comparable to that obtained here. This suggests that even noisy neurofeedback signals can be effective in modulating targeted forms of brain activity.

## 5. Conclusion

Pattern classification analyses play an increasing role in fundamental and applied research settings that make use of neuroimaging. This paper has presented a framework for decoding analysis of EEG data collected in an auditory mismatch paradigm, along with guidelines for the development and optimization of online paradigms based on this framework. Firstly, it demonstrated how MMN data collection and preprocessing can be optimized for pattern classification analysis through careful selection of filter frequencies and appropriate temporal downsampling. The parameters used in these steps were shown to generalize across MMN datasets. It then showed how searchlight, AUC and ERP based methods can be used to evaluate the contribution of components such as the MMN and P3a to single-trial decoding performance. Additionally, estimates of both across-session and across-individual generalization performance were presented. Finally, it was shown how the same decoding methods can be used online to index fluctuations in the amplitude of brain responses at different points in time in single-trial data. This framework can serve as a basis for subsequent research seeking to implement specific online applications based on an auditory mismatch paradigm.

### Conflict of interest statement

The authors declare that the research was conducted in the absence of any commercial or financial relationships that could be construed as a potential conflict of interest.
