# Supplementary material for: Decoding of single-trial auditory mismatch responses for online perceptual monitoring and neurofeedback
Source: Front Neurosci. 2013 Dec 30;7:265. doi: 10.3389/fnins.2013.00265 (PMC3874475; doi:10.3389/fnins.2013.00265)
Supplement: Supplementary file 1 [file DataSheet1.PDF]

Supplementary materials: collection methods for the two datasets

The two datasets analyzed in the article entitled "Decoding of single-trial auditory mismatch responses for online perceptual monitoring and neurofeedback" were collected in two pilot experiments that implemented a neurofeedback loop based on the decoding of single-trial EEG data collected using a mismatch negativity (MMN) paradigm, in which participants view silent film clips while passively listening to auditory sequences containing standard and deviant stimuli. The first experiment ("Dataset 1") was conducted on a single day, and made use of oddball sequences typically associated with MMN recordings (Näätanen 2007). The second experiment was conducted over four days and made use of so-called "optimal" MMN sequences containing 5 different types of deviant stimuli (Näätanen 2004, 2007).

We first report the information specific to each of the two experimental designs before presenting acquisition and data processing methods common to both of the datasets.

Dataset 1: participant information and design

Participants: 14 participants (aged 18-56) reporting normal hearing and with normal or corrected-to-normal vision participated in the experiment. Three of the authors (LS, JM and PD) participated in the experiment. The remaining participants were recruited through either the Radboud University Research participant system or through the Department of Cognitive Artificial Intelligence at the Donders Centre for Cognition. All participants provided informed consent prior to the experiment. The research was conducted with the approval of the ethics committee of the Faculty of Social Sciences at the Radboud University Nijmegen.

Procedure: Participants completed an initial measurement of their frequency discrimination threshold at 500 Hz using a two-alternative forced choice (2AFC) staircase procedure (3-up, 1-down, estimating the 79% correct threshold, Levitt, 1971). Participants then completed four blocks of EEG measurements, the data from which were used to train a quadratically regularized logistic regression classifier that was utilized in the subsequent neurofeedback blocks. Three of these blocks made use of oddball sequences containing a 500 Hz standard stimulus and a deviant stimulus set to the individual participant's frequency discrimination threshold. The final block presented only repetitions of the stimulus at the individual threshold (i.e. no deviants). Prior to the neurofeedback blocks, participants completed another frequency discrimination threshold measurement. Four additional EEG measurement blocks were then completed using the updated frequency discrimination threshold, and with the presentation of neurofeedback based on the output of the classifier on deviant trials. A final frequency discrimination threshold measurement was made thereafter.

Following the completion of these measurements, two or more additional blocks of EEG measurements were made using a 500 Hz standard stimulus and a 600 Hz deviant stimulus, which was expected to elicit large MMN responses given the

salience of the stimulus contrasts. These data serve as the basis of the analyses performed. Two participants (2 and 14) completed three blocks of these measurements, while one participant (1) completed four blocks of measurements.

Stimuli: The stimuli used in the experiment were pure sinusoidal tones with a duration of 100 ms and cosine on/offset envelopes of 5 ms. The fundamental frequency (f0) of the tones was always between 500-600 Hz.

Sequences: EEG measurements were made using oddball sequences containing 85% standard stimuli (always 500 Hz) and 15% deviant stimuli (either threshold or 600 Hz) with a stimulus onset asynchrony (SOA) of 500 ms. Each block included a total of 150 deviant trials. These blocks lasted approximately 8.3 minutes. An additional block was measured prior to the classifier training that included 450 repetitions of the threshold stimulus.

Dataset 2: participant information and procedures

Participants: 12 participants (aged 18-38) reporting normal hearing and with normal or corrected-to-normal vision participated in the experiment. The participants were recruited through either through the Department of Cognitive Artificial Intelligence at the Donders Centre for Cognition (participants 1-6) or through the Radboud University Research participant system (participants 7-12). All participants provided informed consent prior to the experiment. The research was conducted with the approval of the ethics committee of the Faculty of Social Sciences at the Radboud University Nijmegen.

Procedure: Participants completed four sessions of measurements on separate days within a week. At the beginning of the first and the end of the fourth day, participants completed three measurements of their frequency discrimination threshold, and three measurements of their duration discrimination threshold, using the same type of staircase procedure as for Dataset 1. Participants also completed multiple blocks of EEG measurements on each day.

For the first six participants (1-6), two sets of measurement blocks were completed in the first three days: offline, and neurofeedback. Only neurofeedback measurements were made on the fourth day. In the offline measurements, five EEG blocks were measured and used for training a classifier for use in the neurofeedback portion of the experiment. At least five additional EEG blocks were measured in the neurofeedback portion of each session. For the second six participants (7-12), only neurofeedback measurements were made. Neurofeedback was generated using a classifier trained on the first six participant's data. A total of eight EEG blocks were measured in each of the four sessions.

For the analyses, only data from the first three days of measurements was utilized. The first three blocks of data recorded in each session were used in the analyses.

Stimuli: The stimuli used in the experiment were harmonic tones containing two partials at 2*f0 (- 3 dB) and 3*f0 (-6 dB) with a duration of 75 ms and cosine on/offset envelopes of 5 ms. The fundamental frequency (f0) of the tones was always 500 Hz, with the exception of the frequency deviant. Five types of deviant stimuli were used in a design based on Naatanen 2004: frequency (550 Hz), amplitude (±10 dB), duration (25 ms), location (± 800 µS ITD) and gap (25 ms ramped silence inserted between 25-50 ms).

Sequences: Stimuli in the EEG measurement blocks were presented using "optimal" MMN sequences (Naatanen 2004) containing 50% standard stimuli (500 Hz, 75 ms duration) and 10% of each type of deviant. Standard and deviant stimuli constantly alternated with an SOA of 500 ms, with all five types of deviants occurring within the span of 5 deviant trials in pseudorandom order. A total of 300 standard trials and 300 deviant trials were measured in each block. Each block lasted approximately 5 minutes.

Common data acquisition and analysis methods

EEG Data Acquisition: EEG was measured using a 64-channel BioSemi ActiveTwo amplifier and active AgCl electrodes at a sample rate of 2048 Hz. Electrode placement was performed according to the international 10-20 system. Additionally, horizontal/vertical EOG and left/right mastoid leads were recorded. Data acquisition was performed inside an electrically shielded and acoustically attenuated cabin.

Stimulus Presentation: Auditory stimuli were presented via Etymotic ER-P4 insert headphones at approximately 70 dB SPL (excluding loudness deviants used during the collection of Dataset 2) inside an acoustically attenuated cabin using a Macintosh iMac computer via a MOTU 828 mk3 audio interface and the PsychToolbox MATLAB toolkit. Participants viewed silent films on a 15.4" TFT display approximately 60-70 cm from their eyes during the measurements. Films were presented in a 300x400 pixel region centered within the 800x600 pixel display, in order to reduce eye movements to the corners of the screen.

Data Pre-processing: The raw EEG data was pre-processed using the Fieldtrip software toolkit for MATLAB (Oostenveld et al., 2011). Data from all deviant trials and from the standard trials preceding them were selected for analysis. For Dataset 1, this was 30% of all recorded trials. All available trials were used for Dataset 2. Data were initially sliced and stored in epochs ranging from -200 to 600 ms relative to stimulus onset, and resampled to 256 Hz.

A bad-channel detection and repair procedure was then carried out on a per epoch basis. Channels with offsets exceeding ±35 mV or with 50 Hz power exceeding 1000 µV^2 were repaired using a spherical spline interpolation of the neighboring electrodes (Perrin 1989).

Data were then baseline-corrected to the mean of each channel in the 50 ms period prior to stimulus onset, and an independent component analysis (ICA) using all available data from a given recording session was performed. The ICA

was carried out using the infomax algorithm, as implanted by the 'runica' function of the EEGLab toolkit (Delorme and Makeig, 2004). The purpose of the ICA was to remove components from the data containing muscular artifacts such as eye movements (Jung 2000), thus preserving a larger number of trials in the data that would otherwise be lost during the subsequent artifact rejection step. This has been shown to be a useful step when analyzing individual MMN data (Bishop 2009). Only components accounting for greater than 1% of the total variance in the data were considered for removal. The mean variance across epochs was calculated for each component, and the overall mean across components was set as a threshold, with components above the threshold selected for removal. The topography of each component was inspected, and incremental adjustments to the threshold were made on a per participant basis to ensure that non-artifactual components (i.e. those containing auditory responses) were not removed from the data. Following the removal of the selected components, data were reprojected back onto the original EEG channels.

Following the ICA, data were band-pass filtered between 1 and 25 Hz. Epochs with activity in EEG channels exceeding ±75 μV were removed from the dataset. Data were then re-referenced to the average of the left and right mastoid leads.
